# Effect of Non-Concentrated and Concentrated Vaporized Hydrogen Peroxide on Scrapie Prions

**DOI:** 10.3390/pathogens9110947

**Published:** 2020-11-13

**Authors:** Akikazu Sakudo, Risa Yamashiro, Chihiro Harata

**Affiliations:** 1 School of Veterinary Medicine, Okayama University of Science, Imabari, Ehime 794-8555, Japan; 2 Laboratory of Biometabolic Chemistry, School of Health Sciences, University of the Ryukyus, Nishihara, Okinawa 903-0215, Japan; yamashirorisa00@gmail.com; 3 Canon Lifecare Solutions Inc., Minato-ku, Tokyo 108-0075, Japan; chihiro1.harata@medical.canon

**Keywords:** hydrogen peroxide gas, inactivation, medical device, prion, VHP

## Abstract

To date, there have been no studies on the sterilization of prions by non-concentrated and concentrated vaporized hydrogen peroxide (VHP) applied by the same instrument. Here, the effect of the two types of VHP applied using an ES-700 sterilizer on prions was investigated. Brain homogenate from scrapie (Chandler) prion-infected mice was spotted on a cover glass and subjected to ES-700 treatment in soft (non-concentrated VHP from 59% hydrogen peroxide) or standard (concentrated VHP from 80% hydrogen peroxide) mode. Proteinase K-resistant prion protein (PrPres), an indicator of the abnormal isoform of prion protein (PrP^Sc^), was reduced by ES-700 treatment under several conditions: SFT1/4 (soft mode, quarter cycle), SFT1/2 (soft mode, half cycle), SFT1 (soft mode, full cycle), and STD1/2 (standard mode, half cycle). PrPres was detected after the first and second rounds of protein misfolding cyclic amplification (PMCA) of untreated samples, but was undetectable in SFT1/4, SFT1/2, SFT1, and STD1/2 treated samples. In a mouse bioassay, SFT1/2 and STD1/2 treatment of prions significantly prolonged survival time, suggesting that prion infectivity is reduced after ES-700 treatment. In summary, both non-concentrated and concentrated VHP inactivate prions and may be useful for the low-temperature sterilization of prion-contaminated medical devices.

## 1. Introduction

Prion diseases are a group of progressive and ultimately fatal neurodegenerative disorders caused by a proteinaceous infectious prion that can affect both humans and animals. The major component of the pathogen is the abnormal isoform of the prion protein (PrP^Sc^) [1]. Human prion diseases may be caused by infection (e.g., Kuru disease and iatrogenic Creutzfeldt–Jakob disease (CJD)) or inheritance (e.g., Gerstmann-Sträussler-Scheinker syndrome (GSS), familial CJD, and fatal familial insomnia (FFI)), but most types of human prion disease (85–90%) are of unknown origin and are referred to as sporadic CJD [1,2]. All types of prion disease are transmissible [1,2]. Representative animal prion diseases include scrapie in sheep and goat, bovine spongiform encephalopathy (BSE) in cattle, and chronic wasting disease (CWD) in cervids.

When individuals and animals are infected with prions, the cellular isoform of prion protein (PrP^C^), which is expressed in host cells (especially neurons), undergoes a conformational change to PrP^Sc^. The PrP^Sc^ isoform subsequently accumulates in the brain, where it causes neurological symptoms such as dementia, and ultimately leads to death [3]. Importantly, PrP^Sc^ is infectious and can be transmitted to other individuals and animals. Thus, the conversion of PrP^C^ to the pathogenic form is a critical event in prion diseases [4,5]. It is believed that the interaction of endogenous PrP^C^ substrate with the pathogenic PrP^Sc^ template causes PrP^C^ to unfold and refold as PrP^Sc^, a β-sheet-rich isoform of the protein [6]. This conformational change initiates a chain reaction, where each newly converted PrP^Sc^ molecule interacts with more PrP^C^ molecules, fueling the formation of PrP^Sc^ [2,7]. An innovative technique known as protein misfolding cyclic amplification (PMCA) mimics this nucleation-dependent replication process to amplify PrP^Sc^ in vitro [8,9,10] and has been used to evaluate the effect of prion decontamination procedures [11,12,13].

In terms of inactivation, prions are acknowledged to be the most resistant type of pathogen [14]. Conventional sterilization procedures, such as autoclaving at 121 °C for 20 min and exposure to ultraviolet or γ-ray irradiation, fail to inactivate prions [15]. In the case of autoclaving, severe conditions (134 °C for 18 min) are required to inactivate the prion agent [16]. Although treatment with sodium dodecyl sulfate (SDS) and sodium hydroxide (NaOH) can inactivate prions, these procedures are generally regarded as impractical. Furthermore, methods such as immersion in 1 M NaOH or 2% NaOCl, and autoclaving at 134 °C for 18 min may damage some medical devices. 

To clean heat-sensitive instruments such as endoscopes, hydrogen peroxide gas plasma sterilizers (i.e., STERRAD^®^, Advanced Sterilization Products, Johnson & Johnson Company) are widely used [16]. Notably, however, the common cycle conditions of STERRAD^®^ generate the plasma only after exposure and removal of the hydrogen peroxide gas by a vacuum [17]. Furthermore, studies have shown that there is little or no difference in the microbicidal activity of the instrument in the presence and absence of the plasma [18,19,20]. Therefore, the observed antimicrobial effect of STERRAD^®^ is due to vaporized hydrogen peroxide (VHP) and not the plasma itself. 

Indeed, VHP is a low-temperature technology that can also be used for the sterilization of heat-sensitive medical devices [21]. Both non-concentrated and concentrated VHP can be used for sterilization. Hydrogen peroxide concentrations of 30–59% are commonly used to generate VHP under non-concentrated conditions, while concentrated VHP is derived from hydrogen peroxide levels of more than 80% [21]. Although higher concentrations theoretically facilitate increased penetration of the sterilant into difficult to access areas of medical devices, such as lumens [21], hydrogen peroxide concentrations above 60% are less preferable, being dangerous to transport and classified as “Packing Group1” by the US Department of Transportation [22]. Several studies using various VHP sterilizers have evaluated the inactivation effects of VHP against a range of pathogens [23,24,25,26,27,28], including prions [29,30]. To our knowledge, however, no studies have compared the efficacy of concentrated and non-concentrated VHP applied using the same instrument.

These considerations prompted us to investigate the used of concentrated and non-concentrated VHP as a potential method for inactivating prions. Here, the inactivation of mouse scrapie prion by both types of VHP (soft mode (SFT) for non-concentrated VHP and standard mode (STD) for concentrated VHP) was examined by using the VHP instrument ES-700 (Canon Lifecare Solutions Inc., Tokyo, Japan). Western blot analyses and PMCA of PrP^Sc^, as well as animal bioassays, were performed to elucidate whether concentrated and non-concentrated VHP can reduce the proliferation ability and infectivity of the prion agent.

## 2. Results

**Figure 1:**
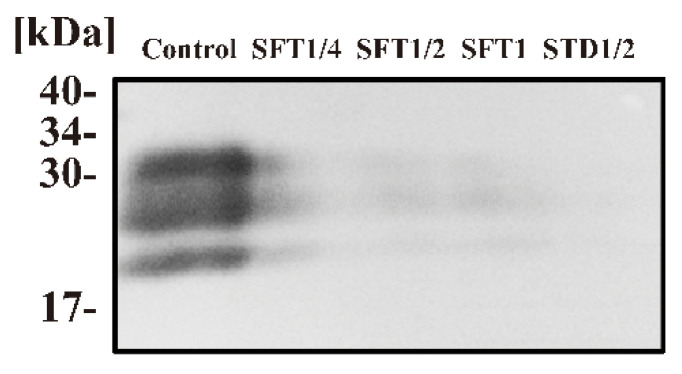
Western blot analysis of prions after processing with ES-700. Prion (Chandler scrapie)-infected mouse brain homogenate was treated with ES-700 in various process modes (SFT1/4 (soft mode for quarter cycle), SFT1/2 (soft mode for half cycle), SFT1 (soft mode for full cycle) or STD1/2 (Standard mode for half cycle)). An untreated sample (Control) was included as a negative control. Samples recovered from treatment spots on the cover glass were subjected to Bio-Rad DC protein assay (Bio-Rad, Hercules, CA, USA) and adjusted to the same protein concentration. Samples (100 μg protein per 50 μL) were then incubated at 37 °C for 60 min in the presence of proteinase K (PK; 20 µg/mL) and subsequently analyzed by sodium dodecyl sulfate-polyacrylamide gel electrophoresis (SDS-PAGE) and subjected to Western blotting using an anti-prion protein (PrP) antibody (SAF83). Molecular mass markers (kDa) are indicated on the left.

First, samples of prion (Chandler)-infected mouse brain homogenate were subjected to ES-700 treatment, followed by proteinase K (PK) treatment and then Western blotting using an anti-PrP antibody (SAF83) (Figure 1). A strong band corresponding to PrPres (an index of PrP^Sc^), which was resistant to PK digestion, was readily detected in the untreated (Control) sample. By contrast, the intensity of the band corresponding to PrPres was lower in all four ES-700-treated samples under various process modes (SFT1/4 (soft mode for quarter cycle), SFT1/2 (soft mode for half cycle), SFT1 (soft mode for full cycle), and STD1/2 (standard mode for half cycle)) than in the untreated sample (control). Analysis using ImageJ software (ImageJ version 1.52a; National Institute of Health, Bethesda, MD, USA) supported the results, which showed relative band intensities of 100% for the control, 28.37% for SFT1/4, 17.70% for SFT1/2, 17.14% for SFT1, and 7.93% for STD1/2. These results suggest that PrP^Sc^ was degraded and/or became sensitive to PK by ES-700 treatment under both concentrated and non-concentrated VHP conditions, even after just a half or quarter cycle.

**Figure 2:**
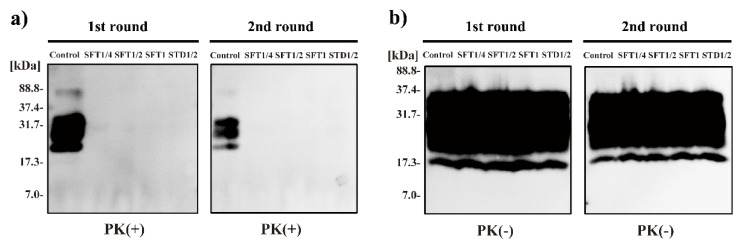
Inhibition of protein misfolding cyclic amplification (PMCA) of scrapie prion by ES-700 treatment. PMCA was performed using normal mouse brain homogenate as PrP^C^ substrate. Untreated (Control) or ES-700-treated [SFT1/4 (soft mode for quarter cycle), SFT1/2 (soft mode for half cycle), SFT1 (soft mode for full cycle), and STD1/2 (standard mode for half cycle)] samples recovered from treatment spots on the cover glasses were diluted 10-fold with PrP^C^ substrate and subjected to a first round of PMCA; a second round was carried out after a further 10-fold dilution with PrP^C^ substrate. An aliquot of each sample was then incubated with PK (20 μg/mL) at 37 °C for 60 min (**a**) or without PK (**b**). Samples were analyzed by SDS-PAGE and Western blot analysis with an anti-PrP antibody (SAF83) to detect PrPres. Molecular mass markers (kDa) are indicated on the left.

Next, the in vitro proliferation ability of prions after ES-700 treatment was analyzed by using PMCA (Figure 2). The amplification efficiency of PrP^Sc^ (Chandler prion) was compared by using uninfected C57BL6/J mouse brain homogenate as a source of PrP^C^ substrate. After ES-700 treatment, samples were diluted 10-fold with PrP^C^ substrate and subjected to a first round of PMCA. This process was repeated on the resulting samples for a second round of PMCA. Aliquots of the samples were treated with PK (PK(+)) or without PK (PK(-)), and then analyzed by Western blotting using anti-PrP antibody SAF83. For the untreated sample, a band of PrPres was detected after the first round of PMCA followed by digestion with PK (PK(+)); however, no such band was detected for the ES-700-treated samples (SFT1/4, SFT1/2, SFT1, STD1/2). Similarly, PrPres was also detected in untreated samples that had been subjected to a second round of PMCA, followed by digestion with PK (PK(+)). By contrast, PrPres was not detected in ES-700-treated samples (SFT1/4, SFT1/2, SFT1, STD1/2) after the second round of PMCA in PK(+). On the other hand, bands of total PrP, mainly corresponding to the PrP^C^ substrate, were similarly detected in all PK(-) samples after the first and second rounds of PMCA. Overall, these results indicate that the in vitro proliferation ability of the prion was reduced by all four types of ES-700 treatment (SFT1/4, SFT1/2, SFT1, STD1/2). These observations suggest that ES-700 treatment with both non-concentrated and concentrated VHP eliminates PrP^Sc^ amplification capability, even after just a half or quarter cycle.

**Figure 3:**
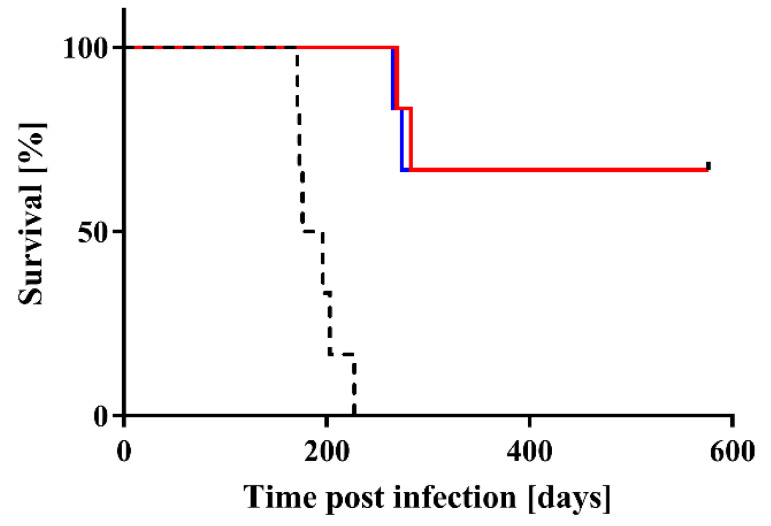
Mice injected with scrapie prion after treatment with ES-700 survived longer than those injected with untreated scrapie prion. Survival curves of mice injected intracerebrally with Chandler prion-infected mouse brain homogenate that was ES-700-treated (red line: SFT1/2; blue line: STD1/2) or untreated (dotted black line: Control) were compared by log-rank test (where *p* < 0.01 was considered significant).

Next, Chandler prion-infected mouse brain homogenate that was ES-700-treated (SFT1/2 and STD1/2) or untreated (Control) was injected into the brains of C57BL/6J mice and the three groups were compared in terms of survival and disease incubation time. The survival curves are shown in Figure 3. All six Control mice displayed abnormal behavior including tremors and ataxia, and died before 227 days. By contrast, none of the mice in groups SFT1/2 and STD1/2 died during this 227-day period. Of the six mice in each of the two ES-700-treatment groups (SFT1/2 and STD1/2), four showed no clinical symptoms of prion disease before 576 days. The other two mice died at 265 and 274 days, and at 269 and 283 days in the SFT1/2 and STD1/2 groups, respectively. Statistical analysis by the log-rank test showed that the survival curve different significantly between the Control group and the SFT1/2 or STD1/2 group. However, there were no significant differences between SFT1/2 and STD1/2. 

Disease incubation time showed a similar trend to survival, with a clear difference between the Control group and the SFT1/2 or STD1/2 group. The mean incubation time of mice injected with untreated prion (191.0 ± 9.0 days) was shorter than that of mice injected with ES-700-treated prion (SFT1/2, >265 days; STD1/2, >269 days) (Table 1). 

**Figure 4:**
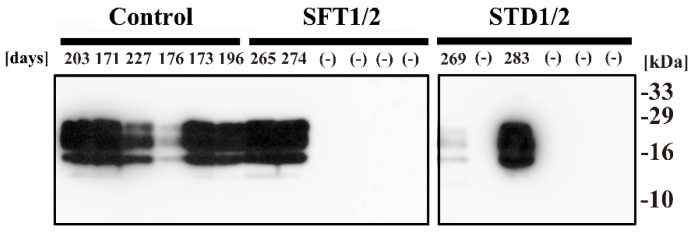
Detection of PrPres in the brain of mice that succumbed to disease. The brain of mice injected intracerebrally with Chandler prion-infected brain homogenate that was treated with ES-700 under SFT1/2 and STD1/2 conditions or untreated (Control) was collected either at the time of end-stage disease (days) when the mice showed clinical symptoms such as tremors and ataxia or at 577 days (–) if the mice survived until 576 days. To determine the presence of PrPres, samples were incubated with PK, and analyzed by SDS-PAGE, followed by Western blotting using anti-PrP antibody SAF83. Molecular mass markers (kDa) are indicated on the right.

Furthermore, PrP^Sc^ accumulation in the brains of mice that succumbed to the disease was confirmed by Western blotting, whereas no PrP^Sc^ accumulation was detected in the brains of mice without signs of scrapie (Figure 4). Namely, the results of Western blotting showed that PrPres was clearly detected in all mice that succumbed to prion disease, including six mice in the Control group and two mice in both groups injected with prions treated with ES-700 (SFT1/2 and STD1/2). These observations demonstrated that all of these mice had succumbed to prion disease, and confirmed that they died due to prion disease. 

Taken together, these results indicate that ES-700 treatment of prion agent under both non-concentrated and concentrated VHP conditions delays the onset of clinical signs of prion disease and prolongs survival. In addition, they suggest that ES-700 treatment decreases the infectivity of the scrapie prion.

## 3. Discussion

Prions are recognized as one of the most difficult biological agents to inactivate by conventional disinfection procedures. Interestingly, liquid hydrogen peroxide is ineffective, whereas VHP efficiently inactivates the prion agent [29]. It is thought that VHP induces protein unfolding and fragmentation, and thereby increases sensitivity to proteolytic digestion by PK, while liquid hydrogen peroxide induces protein clumping, which hinders digestion by proteases [29]. Vacuum conditions may enhance the penetration of hydrogen peroxide and increase the efficacy of prion inactivation [29]. As well as allowing greater penetration of target molecules, the vacuum also augments the reactivity of hydrogen peroxide [29]. The present results, using an ES-700 sterilizer under depressurization with hydrogen peroxide injection, are consistent with those findings.

As mentioned in the Introduction, there are two types of VHP: non-concentrated and concentrated. Although individual non-concentrated and concentrated VHP instruments have been shown to inactivate prions [29,30], no previous studies have evaluated a single instrument applying non-concentrated and concentrated VHP via two modes of operation (SFT and STD). The present study has now shown that prions treated by an ES-700 instrument operating under both non-concentrated and concentrated VHP conditions become highly sensitive to PK treatment and display reduced in vitro proliferation as well as reduced prion infectivity in vivo. 

Sterilization processes can be evaluated in terms of lethality under ‘half-cycle’ conditions using an overkill validation method. For the VHP process, the half-life parameter corresponds to half of the sterilization process time (i.e., VHP exposure time) [31]. In the present study, 4 of 6 (66.67%) mice survived after SFT1/2 or STD1/2 treatment. Thus, more than 50% of mice survived after SFT and STD half cycles, suggesting that one cycle of SFT and STD treatment might further increase the survival rate. Furthermore, after surgical procedures, protein contamination levels are generally considered to range from 8 to 91 µg per instrument [32], whereas the present study used sample material containing approximately 2 mg of protein, suggesting overloaded prion samples. Even in this overloaded condition, however, the inactivation effect of VHP was confirmed with a considerable margin. In addition, before the sterilization process, medical devices are generally subjected to washing with alkaline or enzyme cleaners, which can achieve a 4–6 log reduction in microorganisms and proteins [33]. Thus, this pretreatment combined with ES-700 treatment might increase the total reduction in prion and achieve full inactivation.

The present study used only one prion (Chandler strain), but there are a variety of prions in human and animals. It should be noted that the resistance of prions to inactivation may differ between species or source [34]. Even for the human prion agent, resistance may vary depending on the precise nature of the prion (i.e., variant or sporadic CJD) or whether it is derived from GSS or FFI. Two major types of PrP^Sc^ in human prion diseases have been biochemically characterized, type 1 and type 2 PrP^Sc^ in CJD [35], which may vary in resistance. Sporadic CJD can develop with six genotype/PrP^Sc^ combinations (MM1, MM2, MV1, MV2, VV1, and VV2), resulting in five major strains of sporadic CJD, namely, MM1/MV1, MV2/VV2, MM2 cortical (MM2c), MM2 thalamic (MM2t), and VV1 [36,37,38,39]. Nonetheless, VHP has been shown to be equally effective in vitro against five different strains of prions from human, cattle, hamster, and mouse [40]. These findings suggest that VHP is potentially effective against a variety of prion agents, possibly including all types of animal and human prions. However, further studies will be required to elucidate whether both non-concentrated and concentrated VHP can inactivate all types of prions.

The present study has some limitations. First, the bioassay did not provide quantitative results. Further animal bioassays, including endpoint titration [41] and an incubation time interval assay [42], will be needed to evaluate the extent of the reduction in infectivity caused by ES-700 treatment. Second, the PMCA in the present study was not conducted under optimal conditions and not quantitative. Thus, titration experiments with an increasing PrPres:PrP^C^ ratio in the amplification reaction or quantitative real-time quaking-induced conversion (qRT-QuIC) should be conducted to further analyze the effect of non-concentrated and concentrated VHP on prions. Third, the prion samples were not subjected to simulated soiling, for example, by adding organic matter. Further studies on the effect of soiling during ES-700 treatment are therefore required. Fourth, a cover glass was used as the surface material in this study; however, prions strongly adhere to the surface of some materials. In particular, they have been found to strongly bind to metals [43]. Therefore, the inactivation efficiency of ES-700 treatment against prions may vary depending on the surface properties of the equipment. Fifth, the mouse scrapie prion Chandler strain may not be the best for relevant studies on iatrogenic transmission of prions. Thus, further studies using human prions will be required to evaluate the reduced infectivity of human prions by non-concentrated and concentrated VHP. Sixth, a comparison of non-concentrated and concentrated VHP with other prion disinfectants such as SDS, NaOH, and NaOCl is lacking. Therefore, further comparative studies should be conducted to improve confidence regarding effective or non-effective treatments with data on the sensitivity of the different approaches. Lastly, the nature of the sample material (e.g., brain homogenate, cell lysate, or purified materials) may influence the resistance of prions to disinfection. For example, proteins in the local environment around the prions might reduce the inactivation efficiency of these treatments. Therefore, further studies should be performed to address the efficiency of ES-700 treatment for the inactivation of various prion samples on different types of surface material. 

In conclusion, our findings demonstrate that both non-concentrated and concentrated VHP using the same instrument is an effective method for the inactivation of scrapie prion. As such, this procedure may be invaluable for the sterilization of prion-contaminated medical devices.

## 4. Materials and Methods

### 4.1. Preparation of Mouse Brain Homogenate

Brain homogenates (10% *w*/*v*) were prepared from the brains of terminally diseased C57BL6J mice infected with mouse-adapted scrapie (Chandler strain). Brains were collected, suspended in phosphate-buffered saline (PBS) (Life Technologies, Carlsbad, CA, USA), and then disrupted by passage through a 28-G injection needle before freeze-thawing at −80°C.

### 4.2. ES-700 Treatment of Prions

A 10-µL aliquot of 10% (*w*/*v*) prion-infected mouse brain homogenate, prepared as described above, was spotted onto a cover glass (18 × 32 mm Thickness No.1, 0.12–0.17 mm; Matsunami Ltd., Osaka, Japan) and then air-dried. The cover glass was inserted into a sterilization Tyvek bag (Elk sterilization bag e-TR300; Canon Lifecare Solutions Inc., Tokyo, Japan) and placed in the ES-700 hydrogen peroxide gas sterilizer [44]. The ES-700 carries out the following processes: initial depressurization, sterilization, and alternate ventilation. In the initial depressurization process, the chamber is depressurized from atmospheric pressure to 50 Pa. The sterilization process comprises three steps: depressurization, hydrogen peroxide injection, and sterilization. There are two modes of sterilization: the soft mode (SFT) uses non-concentrated VHP derived from 59% hydrogen peroxide; the standard mode (STD) uses concentrated VHP derived from 80% hydrogen peroxide. Each cycle of ES-700 treatment includes up to four sterilization processes; thus, SFT1/4 (soft mode for quarter cycle) includes one sterilization process under non-concentrated VHP, whereas SFT1/2 (soft mode for half cycle) and SFT1 (soft mode for one cycle) include, respectively, two and four sterilization processes under non-concentrated VHP. STD1/2 (standard mode for half cycle) includes two sterilization processes under concentrated VHP condition. SFT1/4, SFT1/2, SFT1, and STD1/2 were followed by an alternate ventilation process. After treatment, the sample spots were collected with 100 μL of PBS.

### 4.3. Prion Inoculation

Changes in infectivity after ES-700 treatment (SFT1/2 and STD1/2) were assessed by using the recovered samples to intracerebrally inoculate C57BL/6J mice. A 20-µL aliquot of each sample recovered from each spot on the cover glass with 100 μL of PBS was injected into the cerebral ventricular system of each mouse via a microsyringe. Clinical symptoms of prion disease (tremors and ataxia) were monitored over time. The animals were housed according to standard animal care protocols and sacrificed in accordance with University of the Ryukyus guidelines. All experimental procedures were approved by the Animal Ethics Committee of University of the Ryukyus (approval no. A2016002; approval date 7 April 2016).

### 4.4. Western Blot Analysis

Each sample recovered from the treatment spot on the cover glass was subjected to Bio-Rad DC protein assay (Bio-Rad, Hercules, CA, USA) and adjusted to the same protein concentration. Samples (adjusted to 100 μg protein per 50 μL) were treated with 20 μg/mL of PK for 60 min at 37 °C to detect PK-resistant prion protein (PrPres), which is an index of PrP^Sc^ [45]. In some cases, untreated samples (no PK) were included for the detection of total prion protein (PrP), including PrP^Sc^ and PrP^C^. An equal volume of 2× SDS gel-loading buffer (90 mM Tris/HCl pH 6.8, 2% (*w*/*v*) SDS, 0.02% (*w*/*v*) bromophenol blue, 20% (*v*/*v*) glycerol, and 10% (*v*/*v*) 2-mercaptoethanol) was added, and the samples were heated at 100 °C for 10 min to terminate the PK reaction. SDS-polyacrylamide gel electrophoresis (PAGE) using a 12% acrylamide gel was used for protein separation as described previously [46]. The proteins were then transferred to polyvinylidene difluoride (PVDF) membranes (Amersham Biosciences, Piscataway, NJ, USA) by using a semidry blotting system (Bio Rad, Cambridge, MA, USA). The membranes were blocked by incubation for 1 h at 37 °C with 5% (*w*/*v*) skimmed milk (Wako, Osaka, Japan), and then incubated for 1 h at 37 °C with anti-PrP antibody SAF83 (SPI Bio, Montigny le Bretonneux, France) in PBS-Tween (0.1% (*v*/*v*) Tween 20) containing 0.5% (*w*/*v*) skimmed milk. The membranes were washed three times for 10 min in PBS-Tween and incubated with horseradish peroxidase (HRP)-labeled anti-mouse immunoglobulin secondary antibody (Jackson Immuno Research, West Grove, PA, USA) in PBS-Tween containing 0.5% (*w*/*v*) skimmed milk for 1 h at 37 °C. The membranes were subsequently washed three times in PBS-Tween for 10 min. Blots were visualized by using enhanced chemiluminescence (ECL) reagent (Amersham Biosciences), and the signal was detected by using Ez-Capture MG (ATTO Corp., Tokyo, Japan). After Western blot analysis, band intensities were calculated by using image data analysis software (ImageJ version 1.52a; National Institute of Health). Band intensities were determined as a mean field intensity value after adjusting images to a standard threshold value. The band intensity of the untreated sample (control) was set to 100% and the corresponding bands of SFT1/4, SFT1/2, SFT1, and STD1/2 were reported relative to the control.

### 4.5. Protein Misfolding Cyclic Amplification

PMCA of samples recovered from the treatment spots on the cover glasses was performed by using an automatic cross-ultrasonic protein-activating apparatus (ELESTEIN 070-GOT; Elekon Science Corp., Chiba, Japan) [46]. C57/BL6J mouse brain homogenates were used as the source of PrP^C^. Amplification comprised 40 cycles of sonication (3-s pulse oscillation repeated five times at 1-s intervals), followed by incubation at 37 °C for 1 h with gentle agitation in a polystyrene tube (Catalogue No. 035-12; Elekon Science Corp.). Tubes were capped and sealed during the amplification process. The amplified products obtained after the first round of amplification were diluted 1:10 with each PrP^C^ substrate (10% C57/BL6J mouse brain homogenate in PBS containing 4 mM EDTA, 1% Triton X-100, and complete protease inhibitors (Roche Diagnostics, Mannheim, Germany)), and a second round of amplification was performed. After PMCA, the samples treated with or without PK were subjected to Western blotting as described above, using SAF83 for the detection of PrPres and total PrP.

### 4.6. Statistical Analysis

The log-rank test was used to analyse survival curves in the inoculation experiment. The analysis was carried out by using GraphPad Prism 7 software (GraphPad Prism Software Inc., La Jolla, CA, USA).

## Figures and Tables

**Table 1:** Incubation time of mice injected with ES-700-treated prion.

Treatment	Mean Incubation Time ± SEM ^1^	*N*/*N*_0_^2^
Control ^3^	191.0 ± 9.0 days	6/6
SFT1/2	>265 days ^4^	2/6
STD1/2	>269 days ^5^	2/6

^1^ SEM, standard error of the mean. ^2^ *N*, number of dead animals; *N*_0_, number of inoculated animals. ^3^ Control, untreated prion sample. ^4^ Two mice died at 265 and 274 days, while 4 mice survived more than 576 days. ^5^ Two mice died at 269 and 283 days, while 4 mice survived more than 576 days.
